# Hyponatremia and Recurrent Febrile Seizures During Febrile Episodes: A Meta-Analysis

**DOI:** 10.7759/cureus.24398

**Published:** 2022-04-22

**Authors:** Yoshifumi Miyagi, Tomoyuki Sasano, Hiroyuki Kato, Kentoku Kin

**Affiliations:** 1 Department of Pediatrics, Haibara General Hospital, Shizuoka, JPN; 2 Department of Obstetrics and Gynecology, Osaka Saiseikai Nakatsu Hospital, Osaka, JPN

**Keywords:** recurrence, meta-analysis, hyponatremia, febrile seizures, child

## Abstract

Several studies have investigated the potential effects of hyponatremia on recurrent febrile seizures (RFS) during febrile illness. Because findings were inconsistent across studies, we aimed to evaluate the serum sodium levels in febrile seizures (FS) of children with or without RFS during the same episode.

We conducted electronic searches in three databases (PubMed, EMBASE, Cochrane Library) and one scholarly search engine (Google Scholar) up to June 2021 for studies on FS. Screening was done based on the titles and abstracts of primary studies. Then, eligibility was reviewed based on the abstracts. Finally, in order to match the inclusion and exclusion criteria, full-text articles were evaluated by two authors and inconsistencies were discussed. Data extraction was carried out by two independent authors. The extracted variables were author's name, article title, journal name, year of publication, study location, study design, sample size, and mean and standard deviation of blood Na concentration in FS. We performed a risk of bias assessment of included studies using the Newcastle-Ottawa Scale (NOS). The effect size was calculated using the standardized mean difference (SMD), and random-effects models were used for the analysis.

A total of 12 articles were included with a single outlier. This analysis suggested that serum sodium level was lower in patients with RFS during the same febrile episode than in those with single FS, with SMD of -0.70, (n=1784; 95% CI: -1.03, -0.36; Z=-4.10, p<0.01; *I*^2^ 86.67%, p<0.01). In the sensitivity analysis, no significant change was observed in pooled SMD. The optimal cutoff value of serum sodium level was 134.72 mmol/L with an area under the receiver operating characteristic curve of 0.81 (95% CI: 0.61, 1.00), with sensitivity of 80.0% and specificity of 70.0%.

This result indicated a significant association between hyponatremia and RFS during the same febrile episode. Decreased serum sodium levels may be involved in seizure recurrence and may play a role in FS pathogenesis.

## Introduction and background

Febrile seizures (FS) are the most frequent convulsive disorders in children. FS are characterized by episodes of seizures that occur in association with fever in children who do not have an intracranial infection, metabolic disturbance, or a history of afebrile seizure [1]. It has been reported that 14-28% of patients have multiple seizures within the same episode [2,3], and consensus exists regarding the role of serum sodium deficiency as a high-risk factor for seizure recurrence. Several studies have shown that relative hyponatremia can be a predictor of recurrent febrile seizures (RFS) during febrile illness [3-9]. However, other studies have demonstrated that serum sodium levels do not predict RFS during the same febrile episode [10-14]. This study aimed to evaluate the serum sodium levels in FS children with or without RFS during the same episode.

## Review

Methods

Data Sources

Ethical approval was not required because this is a retrospective analysis of previously published data. This study was conducted in accordance with the standard guidelines of the Preferred Reporting Items for Systematic Reviews and Meta-analyses (PRISMA). Two authors conducted electronic searches in three electronic databases (PubMed, EMBASE, and Cochrane Library) and one scholarly search engine (Google Scholar) for eligible studies published up to June 2021.

Study Selection

Patients, who were diagnosed with FS between the ages of 6 months and 6 years, were participants in the study. Previous practice guidelines defined the age of febrile convulsions as 6 to 60 months of age [15], but since we identified several references that included up to 72 months of age, we included 6 to 72 months of age. The exposure was RFS during the same episode. Our study included reports investigating recurrence within 24 hours of the initial seizure in the same category of RFS during the same febrile illness. We adopted single FS as the study control, which is often known as simple FS, but is defined as FS without recurrence; this does not accurately exclude status epilepticus or localized seizures. The principal outcome of this analysis was the difference in serum sodium levels in children with single FS and those with RFS during the same episode. The pooled effect estimate was reported as the standardized mean difference (SMD). All statistical analyses and figures were prepared in R, including the “metafor” package and PyMeta (http://pymeta.com/). Confidence intervals (CI) were set to 95%. Statistical significance was set at a p-value of 0.05. Heterogeneity was evaluated using the *I*^2^ statistic. If *I*^2^ was >50%, a random-effects model was chosen. Sensitivity analysis was performed to assess possible causes of heterogeneity and detect and eliminate outliers. A standard method to detect outliers is to define a study as an outlier if its CI does not overlap with the CI for the pooled effect. The R software function was also used to analyze outliers. Funnel plot analysis was used to evaluate publication bias. Egger’s and Begg’s tests were used to determine publication bias.

Data Extraction

Data extraction was carried out by two independent authors. The extracted variables were author's name, article title, journal name, year of publication, study location, study design, sample size, and mean and standard deviation of serum Na concentration in FS. The following keywords were used in our search strategies: (‘febrile seizures’ or ‘febrile convulsion’) AND (‘hyponatremia’ or ‘sodium’ or ‘natrium’ or ‘electrolytes’) AND (‘child’ or ‘infant’). Potentially eligible articles were assessed for inclusion and exclusion criteria. Studies that met all of the following criteria were included: 1) observational studies that reported FS patients, 2) studies reporting serum sodium level before RFS during the same episode, and 3) studies reporting the presence or absence of RFS during the same episode. Studies that met any one of the following criteria were excluded: 1) conference papers, 2) abstracts, 3) commentaries, 4) letters, and 5) insufficient and inaccurate information. Two independent authors performed a risk of bias assessment of included studies using the Newcastle-Ottawa Scale (NOS) [16].

Results

**Figure 1:**
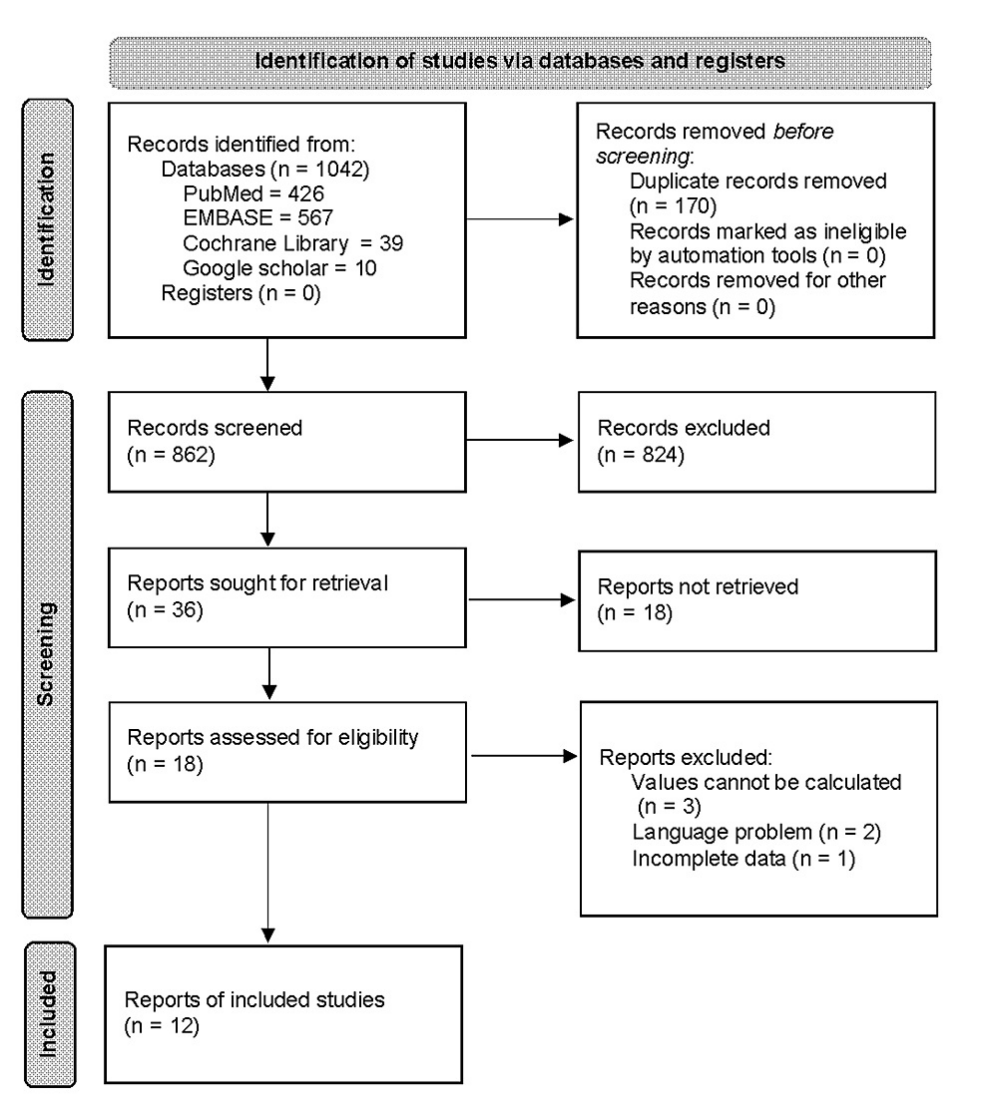
Flowchart of the study selection

A total of 1042 articles were identified from different search tools using the specific search strategies identified with the keywords. Out of 1042 articles, 426 were from PubMed, 567 were from EMBASE, 39 were from Cochrane Library and 10 were obtained from Google Scholar; 862 articles remained following the removal of 170 duplicate reports. The remaining 862 articles were filtered according to the relevance extracted from the abstract for the title and content, after which 18 articles were excluded from this study. After applying inclusion/exclusion criteria with a full-text screen, six articles were excluded. The final 12 reports were found relevant based on the eligibility criteria. A comprehensive PRISMA flow chart is shown in Figure 1 [17].

The results of the two investigators were in agreement. The characteristics of the 11 included studies are listed in Table 1. The sample sizes ranged from 55 to 323, and the reports included 1853 patients aged 6 months to 6 years. Overall, the studies included 415 RFS patients and 1438 single FS patients as controls. Quality assessment was performed using the NOS, as shown in Table 2, and the overall quality was considered moderate. The mean value for the 12 studies assessed was 6.17 or 6.33.

**Table 1:** Characteristics of the studies Salehiomran et al. reported in different units [14].

Author	Year of study	Country	Age group (month)	Sample size (exposure)	Sample size (control)	Serum levels of sodium (mmol/L) (exposure)	Serum levels of sodium (mmol/L) (control)	Significant difference	Study design
Alp and Elmacı [9]	2021	Turkey	12-72	35	209	134.20∓3.55	138.50∓2.38	p < 0.001	Retrospective
Navaeifar et al. [12]	2020	Iran	6-72	66	132	134.35∓2.06	134.46∓2.30	p = 0.333	Prospective
Rahman et al. [6]	2019	Bangladesh	6-60	32	23	135.27∓3.11	138.27∓2.19	Not described	Prospective
Duangpetsang [8]	2019	Thailand	6-60	20	190	130.80∓2.61	132.37∓2.57	p = 0.02	Retrospective
Salehiomran et al. [14]	2018	Iran	6-60	63	208	135∓3 (mg/dl)	136.65∓2.3 (mg/dl)	Not significant	Prospective
Rashied et al. [5]	2017	Iraq	6-60	50	50	131.76∓3.75	135.10∓2.70	P < 0.001	Retrospective
Maksikharin and Prommalikit [11]	2015	Thailand	6-60	47	276	134.49∓3.24	134.94∓3.09	p = 0.41	Retrospective
Nadkarni et al. [7]	2011	India	6-60	21	49	134.30∓3.8	138.20∓3.70	p < 0.01	Prospective
Fallah and Islami [13]	2009	Iran	6-72	20	71	136.55∓4.01	137.37∓4.07	Not significant	Retrospective
Thoman et al. [10]	2004	United States of America	6-60	27	109	135.48∓2.03	135.56∓2.42	p = 1.00	Retrospective
Hugen et al. [3]	1995	Netherlands	6-54	19	50	132.90∓0.40	135.00∓0.50	Not described	Prospective
Kiviranta and Airaksinen [4]	1995	Finland	6-60	15	71	134.20∓2.30	137.62∓2.63	p < 0.01	Not described

**Table 2:** Newcastle-Ottawa Scale scores § was evaluated as a retrospective study and ¶ was evaluated as a prospective study because the type of study was not described clearly [4].

Author	Year of study	Selection	Comparability	Outcome/exposure	Total score
Alp and Elmacı [9]	2021	***	**	**	7
Navaeifar et al. [12]	2020	****	**	**	8
Rahman et al. [6]	2019	**		**	4
Duangpetsang [8]	2019	**	**	**	6
Salehiomran et al. [14]	2018	***	*	***	7
Rashied et al. [5]	2017	****	**	**	8
Maksikharin and Prommalikit [11]	2015	***	**	**	7
Nadkarni et al. [7]	2011	****		*	5
Fallah and Islami [13]	2009	****	*	**	7
Thoman et al. [10]	2004	***	*	**	6
Kiviranta and Airaksinen [4]	1995	**** ***		** *	6 ^§^ 4 ^¶^
Hugen et al. [3]	1995	***		**	5

**Figure 2:**
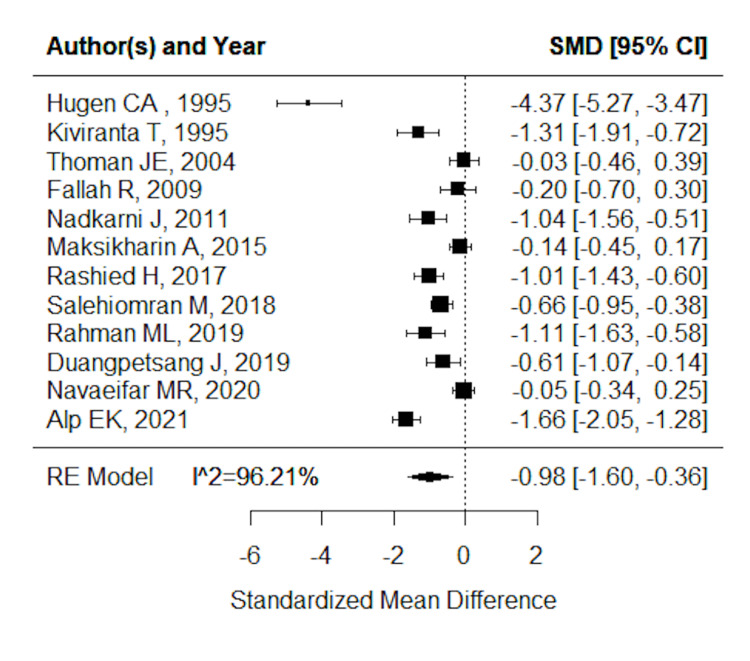
Forest plot of all studies included Hugen CA et al. [3], Kiviranta T et al. [4], Thoman JE et al. [10], Fallah R and Islami Z [13], Nadkarni J et al. [7], Maksikharin A and Prommalikit O [11], Rashied H et al. [5], Salehiomran M et al. [14], Rahman ML et al. [6], Duangpetsang J [8], Navaeifar MR et al. [12], Alp EK and Elmacı AM [9].

Figure 2 shows the forest plot generated for single FS and RFS during the same episode. By conducting a random-effects meta-analysis on the SMD between the single FS and RFS during the same episode, the pooled estimate was -0.98 (n=1853; 95% CI: -1.60, -0.36; Z=-3.10, p<0.01; *I*^2 ^96.21%, p<0.01). We then detected the report by Hugen et al. as the single outlier. Repeating the meta-analysis without this outlier, we obtained a pooled estimate of -0.70 (n=1784; 95% CI: -1.03, -0.36; Z=-4.10, p<0.01; *I*^2^ 86.67%, p<0.01), resulting in a percentage variation of approximately 10% in *I*^2^ (not shown as figure). High heterogeneity indicates unreliability, but it does not appear to be a serious inconsistency, as the effect estimates are in the same direction.

**Figure 3:**
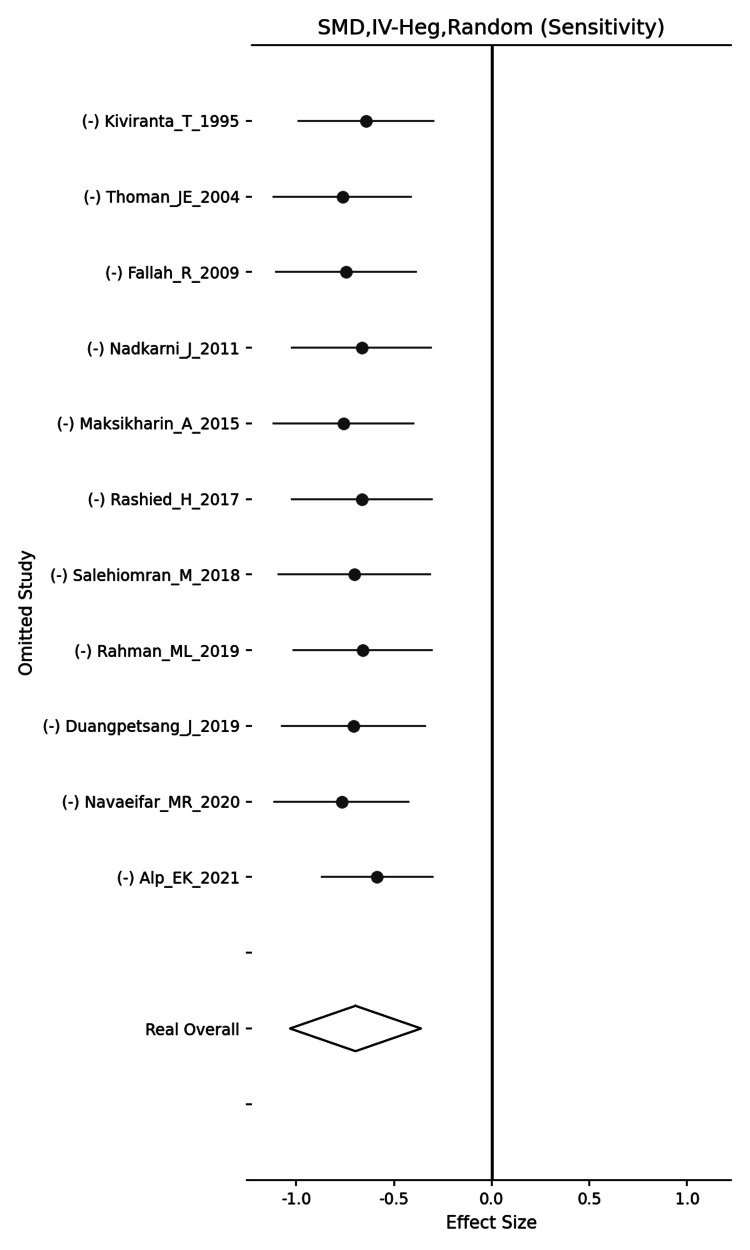
Sensitivity analysis of studies excluding the outlier Kiviranta T and Airaksinen EM [4], Thoman JE et al. [10], Fallah R and Islami Z [13], Nadkarni J et al. [7], Maksikharin A and Prommalikit O [11], Rashied H et al. [5], Salehiomran M et al. [14], Rahman ML et al. [6], Duangpetsang J [8], Navaeifar MR et al. [12], Alp EK and Elmacı AM [9].

By adding a sensitivity analysis after removing the outlier, no significant change was verified in the pooled SMD (Figure 3). Therefore, we concluded that there was a significant association between hyponatremia and RFS during the same episode.

**Figure 4:**
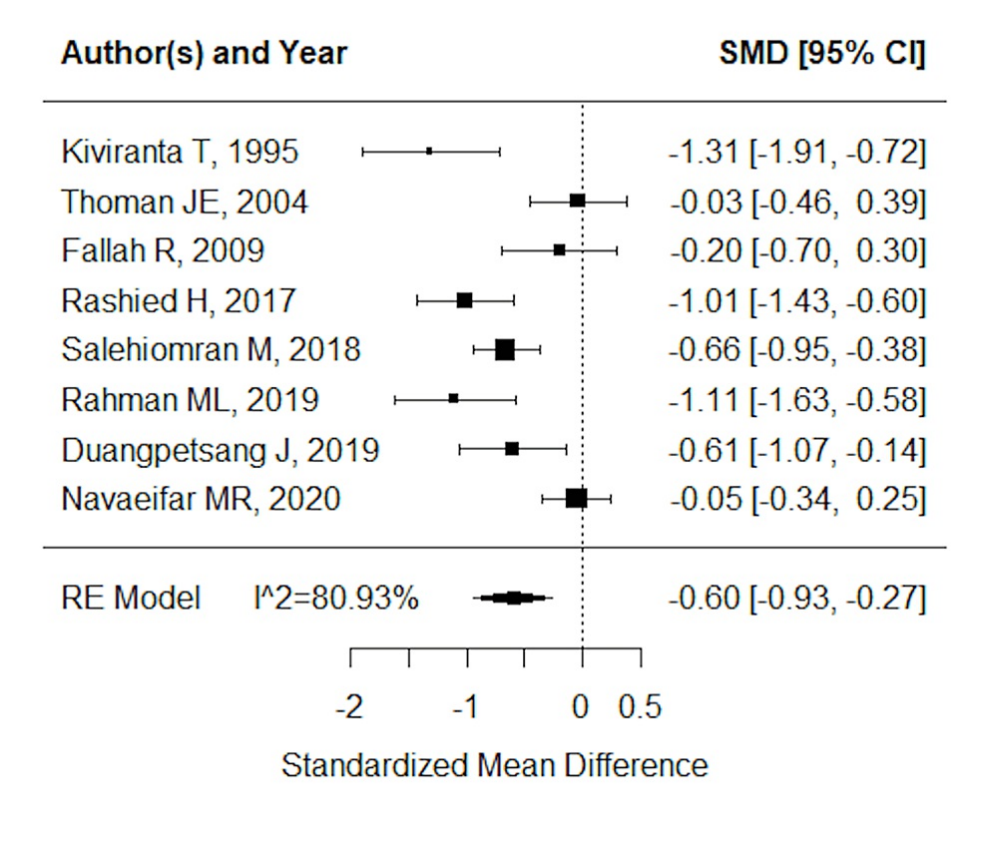
Forest plot of studies using simple febrile seizures as a control Kiviranta T and Airaksinen EM [4], Thoman JE et al. [10], Fallah R and Islami Z [13], Rashied H et al. [5], Salehiomran M et al. [14], Rahman ML et al. [6], Navaeifar MR et al. [12].

We also performed a meta-analysis with simple FS as a control. The results also supported the view that hyponatremia was associated with RFS during the same febrile episode (Figure 4). This suggested that hyponatremia could predict RFS during the same illness even in simple FS, which does not require routine testing.

**Figure 5:**
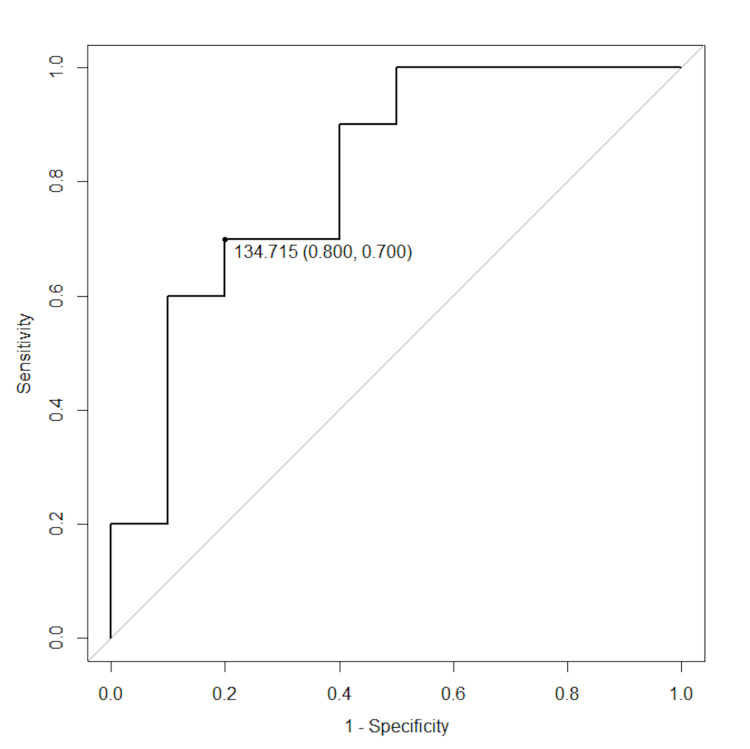
Receiver operating characteristic curve for prediction of recurrent febrile seizures with the same febrile episode based on serum sodium levels

Moreover, we searched thresholds of serum sodium levels that cause RFS during a febrile episode from 10 articles that excluded the outlier and the paper with different units. As shown in Figure 5, the optimal cutoff value of serum sodium level was 134.72 mmol/L with an area under the receiver operating characteristic curve (AUC) of 0.81 (95% CI: 0.61, 1.00) for single FS, with a sensitivity of 80.0% and specificity of 70.0%. The performance of the classifier was moderate.

**Figure 6:**
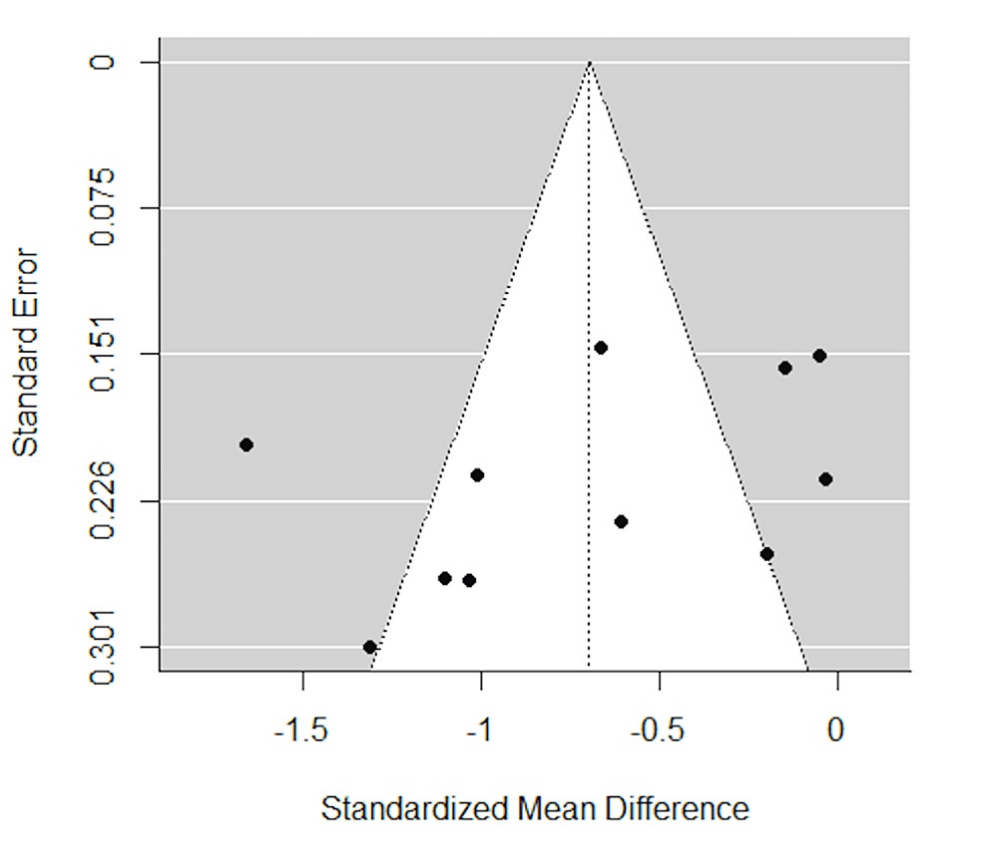
Funnel plot of studies excluding the outlier Egger’s test (p=0.15) and Begg’s test (p=0.21) showed no publication bias.

Finally, the funnel plot against single FS appeared symmetrical (Figure 6). Egger’s and Begg’s tests showed no publication bias (p=0.15 and p=0.21, respectively).

Discussions

This is the first meta-analysis to investigate the relationship between hyponatremia and RFS during the same episode. Moreover, we evaluated the evidence using sensitivity analysis and found the specific reference value for predicting RFS during the same episode. We analyzed 11 observational studies reporting serum sodium levels in 1853 patients with FS. We found that hyponatremia is significantly associated with RFS during the same episode. Studies have identified several underlying factors for FS in healthy children, including a history of antenatal complications [18], zinc deficiency [19], iron deficiency anemia [20], and hypomagnesemia [21]. Lower body temperature (cutoff value 39.2℃) was recently reported as a risk factor for RFS with the same episode (ACU=0.70) [22]. In our report, the AUC was 0.81, which suggests that hyponatremia (sodium level < 134.72 mmol/L) may be a more useful marker than lower body temperature for predicting RFS within the same episode.

The limitations of our analysis need to be addressed. First, our review included retrospective studies. This increases the possibility of bias from numerous possible sources influencing our results. Second, this meta-analysis had high heterogeneity. The random-effects model was applied to weaken the influence of heterogeneity. Moreover, we performed a sensitivity analysis to explore the sources of heterogeneity. Third, articles in non-indexed journals or non-published papers were not searched, thereby introducing some publication bias. Fourth, in this study, the causal relationship was reversed because we used RFS as the exposure and the difference in sodium levels relative to controls as the outcome. What we can also say from the results is that although the serum sodium level is lower in the exposed RFS group than in the control group, we cannot say that hyponatremia causes RFS within the same fever period. Furthermore, the SMD difference in sodium levels is also about -0.5 to -1.0, leaving the possibility that the difference is not physiologically significant.

## Conclusions

In conclusion, our results suggest that hyponatremia is significantly associated with RFS during the same episode. However, it is not possible to conclude from this study whether RFS is affecting hyponatremia or hyponatremia is affecting RFS. Otherwise, the possibility of predicting RFS within the same episode using sodium level < 134.72 mmol/L emerged. As for the causal role of hyponatremia in causing RFS within the same illness, larger prospective studies with exposure as hyponatremia and outcome as RFS within the same fever episode are needed.

## References

[1] Shlomo S (2017). Febrile seizures. In: Swaiman’s Pediatric Neurology.

[2] Berg AT, Shinnar S (1996). Unprovoked seizures in children with febrile seizures: short-term outcome. Neurology.

[3] Hugen CA, Oudesluys-Murphy AM, Hop WC (1995). Serum sodium levels and probability of recurrent febrile convulsions. Eur J Pediatr.

[4] Kiviranta T, Airaksinen EM (1995). Low sodium levels in serum are associated with subsequent febrile seizures. Acta Paediatr.

[5] Rashied H, Sharba S, Hashim JM (2017). The association between hyponatremia and recurrent febrile convulsions. Kerbala J Med.

[6] Rahman ML, Hossain B, Uddin B, Mia SH (2019). Serum sodium level on the recurrence of febrile seizure within the same febrile illness-experience in a district level hospital. TAJ J Teach Assoc.

[7] Nadkarni J, Binaykiya I, Sharma U, Dwivedi R (2011). Role of serum sodium levels in prediction of seizure recurrence within the same febrile illness. Neurol Asia.

[8] Duangpetsang J (2019). Serum sodium levels predict the recurrence of febrile seizure within 24 hours. J Health Sci Med Res.

[9] Alp EK, Elmacı AM (2022). The association between serum sodium levels and febrile seizures recurrence: is the degree of hyponatremia a risk factor?. J Pediatr Neurol.

[10] Thoman JE, Duffner PK, Shucard JL (2004). Do serum sodium levels predict febrile seizure recurrence within 24 hours?. Pediatr Neurol.

[11] Maksikharin A, Prommalikit O (2015). Serum sodium levels do not predict recurrence of febrile seizures within 24 hours. Paediatr Int Child Health.

[12] Navaeifar MR, Abbaskhanian A, Farmanbarborji A (2020). Relation between febrile seizure recurrence and hyponatremia in children: a single-center trial. J Pediatr Neurosci.

[13] Fallah R, Islami Z (2009). Evaluation of serum sodium levels in simple, multiple and recurrent febrile convulsions. Acta Med Iran.

[14] Salehiomran M, Ebrahimzadeh H, Hajiahmadi M (2018). The serum sodium levels and recurrence of simple febrile seizure during the first 24 hours in children. Caspian J Pediatr.

[15] Subcommittee on Febrile Seizures; American Academy of Pediatrics (2011). Neurodiagnostic evaluation of the child with a simple febrile seizure. Pediatrics.

[16] (2022). The Newcastle-Ottawa Scale (NOS) for assessing the quality of nonrandomised studies in meta-analyses. http://www.ohri.ca/programs/clinical_epidemiology/oxford.asp.

[17] (2022). PRISMA. Transparent reporting of systematic reviews and meta-analyses. http://www.prisma-statement.org/.

[18] Sharawat IK, Singh J, Dawman L, Singh A (2016). Evaluation of risk factors associated with first episode febrile seizure. J Clin Diagn Res.

[19] Heydarian F, Nakhaei AA, Majd HM, Bakhtiari E (2020). Zinc deficiency and febrile seizure: a systematic review and meta-analysis. Turk J Pediatr.

[20] Kwak BO, Kim K, Kim SN, Lee R (2017). Relationship between iron deficiency anemia and febrile seizures in children: a systematic review and meta-analysis. Seizure.

[21] Baek SJ, Byeon JH, Eun SH, Eun BL, Kim GH (2018). Risk of low serum levels of ionized magnesium in children with febrile seizure. BMC Pediatr.

[22] Kubota J, Higurashi N, Hirano D (2021). Body temperature predicts recurrent febrile seizures in the same febrile illness. Brain Dev.

